# A Review of the Past, Present, and Future of the Monkeypox Virus: Challenges, Opportunities, and Lessons from COVID-19 for Global Health Security

**DOI:** 10.3390/microorganisms11112713

**Published:** 2023-11-06

**Authors:** Rahim Hirani, Kaleb Noruzi, Aroubah Iqbal, Anum S. Hussaini, Rafay A. Khan, Aleksandr Harutyunyan, Mill Etienne, Raj K. Tiwari

**Affiliations:** 1 School of Medicine, New York Medical College, Valhalla, NY 10595, USA; rhirani2@student.nymc.edu (R.H.); aiqbal6@student.nymc.edu (A.I.); rkhan17@student.nymc.edu (R.A.K.);; 2 Graduate School of Biomedical Sciences, New York Medical College, Valhalla, NY 10595, USA; 3 Department of Pathology, Microbiology and Immunology, New York Medical College, Valhalla, NY 10595, USA; 4 Department of Global Health and Population, Harvard T.H. Chan School of Public Health, Boston, MA 02115, USA; anumhussaini@hsph.harvard.edu; 5 Department of Neurology, New York Medical College, Valhalla, NY 10595, USA

**Keywords:** monkeypox virus, Orthopoxvirus, zoonotic diseases, pandemic, lessons from COVID-19, cross-reactivity, vaccines

## Abstract

Monkeypox, a rare but significant zoonotic and orthopoxviral disease, has garnered increasing attention due to its potential for human-to-human transmission and its recent resurgence in multiple countries throughout Europe, North America, and Oceania. The disease has emerged as a novel threat to the global health systems that are still striving to recover from the major shocks of the COVID-19 pandemic. The unusual manifestation of the illness highlights a substantial knowledge deficit and necessitates the immediate development of a public health action strategy, considering the epidemiological differences observed in the ongoing outbreak and the appearance of cases in non-endemic nations. This literature review aims to synthesize existing knowledge on monkeypox, encompassing its historical context, etiology, epidemiology, surveillance, prevention, transmission, clinical presentation, diagnosis, treatments, and recent outbreak. Particular attention is given to both advances and gaps in our understanding of monkeypox, and we point toward future directions for research and intervention efforts as pertains to vaccine development and distribution. Lastly, we will also review the recent outbreak through a sociopolitical lens as relates to decision-making strategies, especially given the lessons learned from COVID-19.

## 1. Monkeypox Virus: An Introduction

Monkeypox, caused by the monkeypox virus (MPV), an enveloped double-stranded DNA virus, is a seldom-fatal zoonotic disease belonging to the Orthopoxviruses within the Poxviridae family, sharing a 96.3% genome similarity with the variola virus responsible for smallpox [1,2]. MPV was first identified in 1958 in research monkeys originating from Singapore in a Danish laboratory [3]. However, uncertainty remains as to the natural history of MPV [1,3,4]. Despite its name, the virus primarily resides in smaller mammals and rodents, including rats, mice, squirrels, prairie dogs, and monkeys [5]. Transmission to humans remains poorly understood, with aerosol, indirect, and direct contact with live or deceased animals all implicated [1,6]. In the 1970s, MPV cases emerged across six African countries, namely, Cameroon, Côte d’Ivoire, the Democratic Republic of Congo, Liberia, Nigeria, and Sierra Leone, with an initial 48 cases escalating to over 800 cases in the Democratic Republic of Congo [7,8].

The increasing incidence and prevalence of monkeypox in Africa can be attributed to the waning cross-protective immunity in the population following the discontinuation of smallpox vaccination in the 1980s [6]. Studies indicate that the smallpox vaccine provides approximately 85% protection against monkeypox [9]. While the genomic similarities between MPV and variola virus are notable, they exhibit significant differences in the regions of the genome associated with virulence and host range factors [2]. The rising occurrence of MPV can also be linked to the growing proportion of individuals who have never received the monkeypox vaccine, estimated to be over 70% of the population [10].

Although monkeypox and smallpox are not identical, their familial relation has facilitated sufficient cross-immunity [6]. During outbreaks, individuals who previously received the smallpox vaccine displayed milder rash symptoms, were less likely to develop lymphadenopathy, and saw no mortality [11]. Cases were primarily limited to African countries from 1980 to 2000, but as the new century dawned, the disease spread widely across the continent, with the Democratic Republic of Congo reporting 20,000 suspected cases. In 2003, the first monkeypox case outside of Africa was reported in the United States, involving 48 cases linked to prairie dogs infected by imported Gambian pouched rats, demonstrating rodent-to-host transmission [12].

Increased global travel and importing of goods from Africa have contributed to sporadic outbreaks [8]. Cases have been reported in various countries, including one in Israel (2018), seven in the United Kingdom (2018–2021), one in Singapore (2019), and two in the United States (2021) [8]. However, monkeypox remains prevalent in African countries, as evidenced by the 650 confirmed cases in Nigeria from 2017 to 2022, resulting in nine fatalities [13]. The number of cases has surged globally, with approximately 50,000 confirmed cases reported across 100 countries as of August 2022, with 13 reported deaths [14]. This alarming increase in cases led the World Health Organization to declare the monkeypox outbreak a global health emergency [15,16]. While MPV is less fatal and transmissible than the smallpox virus, concerns persist regarding the possibility of it evolving into a more efficient human pathogen [11]. As of October 2022, more than 75,000 cases of MPV were reported across 109 countries, with 34 deaths reported in 12 countries in the year 2022 [17].

In the context of MPV, two distinct clades exist: Central African (clade I) and West African (clade II). Clade I, also referred to as the Congo Basin clade, exhibits higher virulence, with a fatality rate of 10.6%, in contrast to the 3.6% fatality rate associated with clade II [4]. Clade I is further characterized by elevated rates of morbidity, mortality, human-to-human transmission, and viremia [2]. Comparative virulence assessments have been conducted in non-human primates, specifically cynomolgus monkeys, using varying doses of Central and West African strains [18]. Monkeys exposed to high doses of the Central African strain experienced 100% mortality, while those receiving low doses exhibited pronounced morbidity. Conversely, monkeys exposed to either dose of the West African strain all survived, with minor morbidity. Notably, cases reported outside of Africa predominantly involve the West African clade, implying a more favorable prognosis for affected individuals [19]. Recent studies have delved into the molecular epidemiology of these viruses, revealing a high rate of genetic variance and accelerated evolution [20].

## 2. Mechanism of Cell Entry and Viral Replication

The specific mechanisms involved in monkeypox virus’s entry and replication may vary depending on the host cell type and species. Additionally, the virus has evolved to evade host immune responses, further complicating the infection process. Understanding the detailed mechanisms of monkeypox virus’s entry and replication is an active area of research, and new insights may continue to emerge.

In the context of MPV infection, four distinct viral proteins have been identified as integral components facilitating the attachment of MPV to the host cell, as extensively elucidated by Kaler et al. [21]. The viral binding process involves the interaction of MPV with approximately 11 to 12 transmembrane proteins, which are devoid of glycosylation and have a molecular weight ranging from 4 to 43 kDa [21,22]. Notably, certain poxviruses have been reported to employ laminin and heparin sulfate to augment the attachment process [10,23]. Upon successful attachment and entry into the host cell, MPV initiates DNA synthesis, a critical step that transpires within a concise timeframe of no more than 2 h [24]. Subsequently, MV uncoats within the cytoplasm, as a prerequisite for replication. To ensure uninterrupted viral propagation, MPV efficiently incapacitates the host cell’s defense mechanisms through the concerted action of prepackaged viral proteins and enzymatic factors [25]. In the ensuing phases of viral replication, MPV orchestrates the synthesis of early messenger RNA (mRNA) via its DNA-dependent RNA polymerase. The translated early mRNA serves multifarious functions, including facilitating further uncoating, DNA replication, and the generation of intermediate transcription factors [23,25]. Progressing further along the viral life cycle, transcription and translation of intermediate mRNA species transpire, thereby promoting the expression of late mRNAs. Subsequently, the translation of late mRNAs yields both structural and nonstructural proteins. These translated proteins, along with concatenated DNA molecules formed during earlier replication stages, congregate and coalesce to form immature virions (IMVs). These IMVs subsequently mature into MPV and, devoid of an external membrane, initiate infection upon their liberation following host cell disruption [23,26]. Post-formation, these MPV entities undergo intracellular transport facilitated by microtubules, eventually culminating in their fusion with the inner cell membrane to form cell-associated virions (CEVs). The interaction with CEVs triggers actin polymerization and filament development. Finally, these CEVs exit the host cell, now referred to as extracellular enveloped virions (EEVs), marking the culmination of the MPV life cycle [23,27].

The incubation period of MPV is not contagious and has a silent clinical manifestation. It is during the prodromal stage when secondary viremia arises via lymphoid organs to the skin and other tertiary organs, including the eyes, lungs, etc. This is followed by the presentation of symptoms, such as lymphadenopathy, mucocutaneous lesions, etc., making the individual highly infectious during this stage [20].

## 3. Clinical Presentation

The clinical presentation of monkeypox consists of three distinct phases: incubation, prodrome, and rash [28]. Following an incubation period of 7–14 days, the 2-day prodrome phase manifests with fever (38.5–40.5 °C), chills, myalgia, sore throat, and headaches [2,28]. Notably, lymphadenopathy in the maxillary, cervical, and/or inguinal regions, with inguinal involvement being predominant, occurs in approximately 90% of patients [29]. This lymphadenopathy is characteristic of monkeypox [2]. After the prodromal phase, 0.2 to 1.0 cm lesions and rashes initially appear on the face and oral mucosa, subsequently spreading centrifugally over the body [2], marking the period of highest contagiousness [30]. Oral mucosal involvement can lead to pain and difficulty with oral intake in the later stages [16]. Disease severity is correlated with lesion count, with higher counts indicating an elevated risk of complications, such as ocular infections, respiratory depression, encephalitis, and septicemia [12,31].

A systematic review of MPV cases from 2003 to 2021 revealed confusion, seizures, and encephalitis as symptoms in 2% of cases [10]. Lesions progress through macular, papular, vesicular, pustular, and crusted stages before becoming noncontagious [32]. These lesions are prone to bacterial superinfections, particularly in individuals who are not vaccinated against smallpox [29]. Certain population subgroups exhibit distinctive manifestations. Men who have sex with men may present with anal ulcers and painless genital lesions that spread [33,34]. Vertical transmission of MPV can occur in pregnant individuals, often leading to adverse outcomes, including spontaneous abortions and stillbirths characterized by fetal vesicular lesions, hepatomegaly, and hydrops [35,36]. High viral loads are found in fetal tissue, the umbilical cord, and the placenta due to the virus’s ability to breach the syncytiotrophoblast barrier [36]. Young children aged 0–4 years, have a higher fatality rate (14.9%) compared to children over 10 years old (0%). Young children were most affected in the 1970s and 1980s, but the median age of cases has since risen to around 39 years old. Further research into pediatric cases may aid in preventing future fatalities in this demographic.

It is also possible for individuals who are infected with MPV to be asymptomatic [37]. A systematic review and meta-analysis demonstrated that 10.2% of monkeypox cases were asymptomatic or clinically silent. These cases were found to either be asymptomatic at the time of testing and develop symptoms later, to not develop symptoms at all, or to have very mild symptoms that were not noticed by the infected individual [37]. MPV has been isolated from urethral and anal samples of asymptomatic individuals, suggesting that asymptomatic cases are able to transmit the virus [37,38]. However, no case of MPV transmission from an asymptomatic individual to a close contact has been definitively confirmed [37]. Future surveillance and research should aim to continue tracking asymptomatic cases and their close contacts to characterize the probability of MPV transmission from asymptomatic individuals.

## 4. Transmission and Cross-Reactivity

The natural reservoir of MPV remains unidentified, but its transmission to humans typically occurs through intermediate hosts, such as small rodents and mammals [39]. The exact mechanisms of MPV’s transmission to humans have not been definitively established; however, it is known that handling infected animals can lead to human infections, subsequently facilitating human-to-human transmission [40]. Person-to-person transmission primarily occurs through direct contact with infectious lesions on the skin, oral mucosa, or genitals, which may result from activities such as skin-to-skin contact, vaginal/anal intercourse, oral–genital contact, mouth-to-mouth kissing, and even face-to-face conversation due to aerosol transmission [39,41]. Once airborne, the virus can infiltrate the respiratory epithelium and subsequently spread via the lymphatic system to infect major organs [2]. However, it is important to note that the evidence on whether the monkeypox virus can be airborne is still lacking. For smallpox transmission, the virologist Donald Milton noted that the natural infection occurred through large droplets deposited on the mucous membranes of the upper respiratory tract, which had the potential to increase the risk of viremia and death compared with nasal inhalation. Hence, MPV has also been noted to transmit through saliva, and the CDC has also acknowledged the difference between airborne transmission and transmission through respiratory secretions [42,43].

Transmission of MPV can also occur among close contacts, household members, and sexual partners. Notably, sexual transmission, including through semen, has been documented, with a recent 2022 report highlighting cases of monkeypox transmission among men who have sex with men (MSM) engaged in condomless sex [34]. Real-time PCR analysis confirmed the presence of MPV DNA in the semen of infected individuals who remained asymptomatic. This observation underscores the increasing relevance of human-to-human transmission compared to transmission from infected animals, as seen in endemic regions [1]. Recent outbreaks have disproportionately affected MSM with numerous sexual partners [34], with a global case series from April to June 2022 indicating that 98% of cases occurred among gay or bisexual men [44]. Furthermore, MSM exhibited distinct immune profiles in the rectal mucosa compared to heterosexual individuals, characterized by heightened immune activity and evidence of mucosal injuries, which are potential sites for pathogen entry [45,46].

While the current evidence suggests that repeated reintroduction of MPV is necessary to sustain the disease, there is no conclusive proof that human-to-human transmission alone can maintain monkeypox infections within the population [6,11]. Further research is imperative to explore the presence of MPV in bodily fluids like semen and its role in disease transmission, given the limited sample size of affected patients. Understanding the dynamics of MPV transmission among humans is critical for effective disease control and prevention.

The genomes of Orthopoxviruses exhibit remarkable conservation, leading to a significant extent of cross-reactivity and cross-protectivity among antibodies [47]. However, when compared to the other antigens, the prevalent A29 and M1 antigens facilitated more robust cross-neutralizing immune responses against MPV, making them potential antigen candidates for innovative orthologous Orthopoxvirus vaccines [48]. Although there is similarity in the genomes, MPV possesses genes that are either missing, altered, or shortened in vaccinia, and these gene products can serve as discriminative markers to differentiate between monkeypox and vaccinia infections, which could help develop novel diagnostic strategies catered to better detect MPV [47]. For example, utilizing cross-adsorption, Dubois et al. established a quantitative post-adsorption ELISA method to differentiate between monkeypox and vaccinia infections [47]. Notably, even in the challenging scenario of diagnosing clinically silent monkeypox cases among individuals with prior vaccinations, this approach demonstrated an exceptional performance, achieving 100% sensitivity and 100% specificity in the accurate identification of clinically evident monkeypox infection. As noted earlier, the incubation phase, which lasts roughly 1–2 weeks, is not contagious. However, in 2022, a study conducted in the United Kingdom to study the transmission dynamics of MPV indicated that a substantial 53% of monkeypox cases in the ongoing global outbreak are transmitted 1 to 4 days before symptoms appear [49].

As observed during the COVID-19 pandemic, the amalgamation of antigen detection assays with molecular assays plays a pivotal role in mitigating the transmission of infectious diseases. The distinctive attributes of antigen-detecting immunoassays, such as rapid result generation spanning from minutes to hours and their adaptability to lateral flow configurations, render them versatile tools suitable for deployment in diverse settings, including point-of-care facilities, residences, and field environments. Similarly, several antigen detection immunodiagnostic assays have been produced to provide access to better viral detection, ensuring early diagnosis and treatment [50,51].

## 5. A Potential Public Health Crisis: What Is Yet to Be Discovered about MPV?

**Figure 1:**
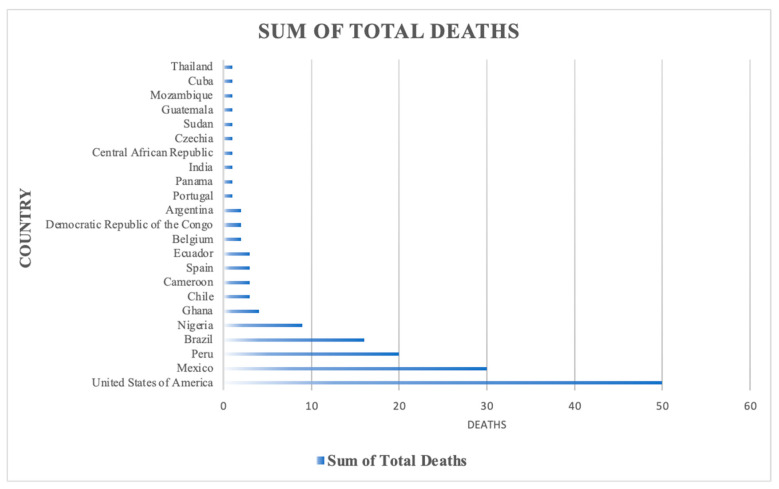
Mortality data from the most recent MPV outbreak. Data were retrieved from the World Health Organization (https://worldhealthorg.shinyapps.io/mpx_global/_w_ab623397/#section-global-deaths-1, accessed on 1 October 2023).

In 2022, 42 nations in five WHO regions reported 2103 laboratory-confirmed cases of MPV, with one reported death between 1 January and 15 June 2022 [52]. As of September 2023, a total of 90,618 cases have been reported from the 2022–2023 MPV outbreak, with 15 countries comprising 80,662 of these cases (Table 1). Moreover, most of the deaths during this recent outbreak were in the USA (Figure 1). Since May 2022, around 98% of documented cases globally have been in MSM [15,52,53]. However, recent cases have not demonstrated a traditional disease pattern. In endemic regions, 20% to 42% of patients have presented with more than 100 lesions, while during the 2022 outbreak only 0 to 4% of the cases presented with more than 100 lesions [54].

The current outbreak differs from the previous ones with respect to age, as most affected cases are in their thirties (instead of affecting young children as in the past), and sex, as most cases are males [8,52]. During previous outbreaks, the location of body rashes included the face, trunk, and limbs, while during the 2022 outbreak lesions were mostly confined to genitals [54]. In 2003, a cluster of 37 patients in the United States had rash as a predominant symptom that lasted for 7 to 24 days (median: 12 days), accompanied by other symptoms such as chills, myalgia, headache, lymphadenopathy, and fever that lasted between 2 and 13 days (median: 8 days) [55]. However, in the current outbreak, rash has been reported as the primary symptom at the disease’s inception, and prodromal symptoms are not always noted. Moreover, most cases of MPX reported in the current outbreak do not have any linkages with travel to an endemic area [55]. The atypical presentation of the disease suggests a significant knowledge gap and calls for an urgent public health action plan, considering the epidemiological variations during this current outbreak, emerging cases in non-endemic countries, and its lack of correlation with human or animal travels.

MPV has been demonstrating non-traditional patterns during the current outbreak. Hence, the Centers for Disease Control (CDC) recommend three-tier protective measures including contact, droplet, and airborne precautions to limit the transmission [56]. Furthermore, patients are recommended to isolate themselves for up to 4 weeks until all skin lesions have crusted over [57]. The examination of monkeypox virus genomes through phylogenetic analysis revealed a notable and statistically significant divergence, alongside emerging subclades during the monkeypox outbreak of 2022. It was observed that the frequency of nucleotide transitions has been on the rise in recent years, possibly attributable to the activity of the apolipoprotein B mRNA-editing enzyme catalytic polypeptide 3G (APOBEC3) deaminase. This microevolutionary trend is likely indicative of the virus’s spread within the community and its ongoing adaptation to the human host [58].

## 6. Vaccine Development and Distribution

Viruses within the Orthopoxvirus (OPXV) genus, such as MPV, variola virus (VARV), vaccinia virus (VACV), and cowpox virus (CPXV), have highly conserved genetic sequences and common antigenic features [24,59,60]. Due to these similarities, infection or immunization with one of these viruses confers cross-protection against other viruses within the OPXV genus [60]. The cross-protective phenomenon underpinned the utilization of live vaccinia virus (VACV) derived from VACV-infected animal lymph to produce the initial smallpox vaccines, Dryvax and Lancy-Vaxina [61,62,63]. Dryvax, derived from the New York City Board of Health (NYCBH) VACV strain and approved by the FDA, was administered within the United States. Conversely, Lancy-Vaxina, derived from the Lister VACV strain, was used internationally [61,62]. These vaccines played a pivotal role in the successful eradication of smallpox [61]. It is important to note here that global commitment, along with the distinctive properties of these vaccines, such as advances in technological innovations to produce heat-stable, freeze-dried vaccines capable of surviving at tropical temperatures without refrigeration, along with the production of easy-to-use and cost-effective bifurcated needles to administer doses, contributed to successful vaccination campaigns in high-risk countries, which were part of the core basis of smallpox’s eradication [64]. Despite their efficacy, the first-generation vaccines were associated with rare but severe adverse events [61,62,63,65,66,67,68]. These vaccines consisted of live, replication-competent VACV [63,65], which likely contributed to the occurrence of adverse effects due to VACV’s replication and spread within the vaccinees. Adverse events encompassed progressive vaccinia, eczema vaccinatum, generalized vaccinia, post-vaccinal encephalitis, myopericarditis, inadvertent autoinoculation, transmission to close contacts, and fatalities [61,62,63,65,67,68].

Progressive vaccinia (PV) primarily afflicted immunocompromised individuals, characterized by improper healing at the vaccination site and persistent VACV replication, resulting in necrosis, secondary metastatic necrotic vaccinia lesions, and potential fatality if untreated [62]. Eczema vaccinatum (EV) manifested as cutaneous dissemination of VACV, leading to diffuse and erosive skin lesions, fever, and lymphadenopathy. Those with atopic dermatitis or eczema faced a heightened risk, with the condition proving fatal in cases of extensive viral dissemination and bacterial superinfection [62,68]. Generalized vaccinia (GV) presented as a diffuse vesicular or pustular rash, likely due to hematological dissemination, with potential severity in immunocompromised individuals [62,68]. Post-vaccinal encephalitis resulted from central nervous system parenchymal inflammation after vaccination, often leading to death or permanent neurological deficits, particularly in infants under one year old [62,68]. Myocarditis and pericarditis were caused by post-vaccination inflammation of the heart or pericardium and could manifest with mild-to-severe chest pain, dyspnea, palpitations, and arrhythmias. It remains unclear whether these conditions were attributed to direct VACV infection or the inflammation associated with the immune response [68]. Inadvertent autoinoculation occurred when vaccinees accidentally transferred VACV from the vaccination site to other parts of their body, frequently the face and genitalia, resulting in pustular or vesicular lesions that eventually crusted over. Similarly, vaccinees could transmit VACV from the vaccination site or lesions to close contacts, either through direct contact or through fomites [62,68]. Infected contacts could subsequently experience any of the reported adverse effects. Ocular inoculation could result in loss of vision [68]. Individuals with compromised immune systems, those with severe autoimmune disorders, and infants under one year old faced an elevated risk of adverse effects that were often more severe in nature. Furthermore, individuals with dermal disruptions due to burns, seborrheic dermatitis, psoriasis, or severe acne were at an increased risk of adverse effects [62]. Finally, the vaccination of pregnant women led to vertical transmission to the fetus, resulting in premature birth, infant skin lesions, scarring, or fetal and neonatal mortality [62,68,69,70]. While relatively rare, the severe adverse events associated with first-generation smallpox vaccines were deemed unacceptable, leading to their discontinuation after the successful eradication of smallpox. Subsequent research efforts have focused on the development of safer smallpox vaccines [63,66].

The second-generation smallpox vaccine, ACAM2000, was developed from a live, purified clone of the same vaccinia strain used in Dryvax, and in 2017 it was approved by the FDA and recommended by the Advisory Committee on Immunization Practices (ACIP) for use in individuals at high risk of infection. ACAM200 was subsequently determined to have a safety profile similar to that of Dryvax, including risk of myopericarditis, encephalitis, progressive vaccinia, and Stevens–Johnson syndrome, and research efforts to develop a safer smallpox vaccine continued [71,72]. JYNNEOS is a third-generation smallpox vaccine that was licensed by the FDA in 2019 for individuals who are 18 years old or older. It was developed from modified vaccinia Ankara (MVA), which is a VACV strain that is incapable of replication. Numerous studies have confirmed the robust immunogenicity and safety of JYNNEOS vaccination [73,74,75]. Common adverse effects primarily involve local symptoms, including pain, tenderness, erythema, and induration at the vaccination site, as well as systemic effects such as fever, fatigue, lymphadenopathy, headache, and myalgia [74,76,77]. These adverse effects are consistent with those seen in other live attenuated vaccines like intranasal influenza, MMR, and varicella vaccines. Notably, no serious adverse events associated with Dryvax and ACAM2000, such as PV, EV, GV, post-vaccinal encephalitis, or myocarditis, have been reported after JYNNEOS vaccination [73,74,75]. While one case of possible pericarditis was reported, it was likely unrelated to JYNNEOS, as the subject tested positive for coxsackie B virus. Rare cardiac events like bundle-branch block, palpitations, and tachycardia have been reported but are infrequent [74,75]. A clinical trial demonstrated that JYNNEOS is not inferior to ACAM2000 in terms of immunogenicity, with fewer adverse events [76]. Studies comparing JYNNEOS in healthy individuals and those with HIV or atopic dermatitis show similar immunogenicity and safety profiles, suggesting its potential use in immunocompromised and atopic dermatitis populations. The ACIP recommended JYNNEOS for high-risk individuals working with OPXV or involved in healthcare. The sole contraindication is a severe allergy to one of its components [72].

Despite JYNNEOS’ favorable safety profile, its use in pregnant and breastfeeding women and those under 18 years of age remains uncertain. Historically, smallpox vaccines with replication-competent VACV posed risks to pregnant women and fetuses. However, there are insufficient data on JYNNEOS in this context. It is unlikely that JYNNEOS can be transmitted to the fetus or through breastmilk due to its replication-deficient MVA composition, as supported by animal studies [72]. Furthermore, JYNNEOS lacks the adverse effects seen with replication-competent VACV vaccines. It is potentially suitable for pregnant and breastfeeding women when clinically indicated [72]. JYNNEOS is not approved for use in individuals under 18 years of age, although limited experiences in young children and infants in the UK and with the same VACV strain in other experimental vaccines show no serious adverse effects. JYNNEOS has been authorized for use as post-exposure prophylaxis in patients under 18 years of age during an outbreak [78]. Continued monitoring and analysis of JYNNEOS vaccination in pregnant women, breastfeeding women, and those under 18 years of age will be essential to assess the risk of adverse events in these populations. The Centers for Disease Control (CDC) currently recommend immunization against MPV with JYNNEOS for individuals who have had known or suspected exposure to MPV, have had sexual intercourse in a commercial sex venue or in exchange for money or items in the past six months, or are MSM, transgender, non-binary, or gender-diverse individuals who have been diagnosed with one or more sexually transmitted infections (STIs) or have had sex with more than one partner in the past six months [79].

JYNNEOS is administered as two doses spaced four weeks apart [72]. There is still some uncertainty regarding the duration of immunogenicity and the optimal schedule of booster administration following vaccination with the primary series. An initial clinical trial revealed seroconversion rates of 100% after two JYNNEOS doses, with a subsequent decrease to 72.2% at 30 weeks and 66.7% after one year. A booster dose 12 weeks after the primary series increased rates to 95.8% [80]. A recent 2022 study reported declining seroconversion rates of 89.2% at six weeks, 65.2% at 30 weeks, and 5.4% at two years after two JYNNEOS doses, highlighting waning immunity. A booster two years after the primary series resulted in rates of 92.0% after 1 week and 88.7% at 30 weeks [77]. Both studies support strong B-cell memory responses with a booster after two initial doses. Future research should investigate optimal booster intervals to maximize immunogenicity in high-risk individuals.

Administration of two doses of JYNNEOS confers greater protection against OPXV than the administration of one dose [81]. Among the 1,189,651 doses of JYNNEOS administered in the United States from May 2022 to January 2023, 61.7% were first doses, 38.1% were second doses, and 0.2% were dose three or higher [82]. There were fewer second doses administered than first doses, indicating that many individuals did not receive a second dose and were not optimally immunized against OPXV [76]. Ensuring that individuals follow up and receive both doses of the vaccine should be prioritized moving forward to maximize immunization against MPV and prevent future outbreaks of MPV.

The U.S. Department of Health and Human Services (HHS)’s Administration for Strategic Preparedness and Response (ASPR) currently distributes JYNNEOS based on a jurisdictional threshold approach. The threshold is defined as the estimated percentage of the national population within a jurisdiction for whom JYNNEOS vaccination is currently recommended by the CDC [82,83]. Each jurisdiction is able to order vials of the JYNNEOS vaccine until they reach their respective threshold [83]. The JYNNEOS vaccination coverage is defined as the estimated percentage of the national population for whom vaccination is recommended that was actually vaccinated. From May 2022 to January 2023, the vaccination coverage was 36.7% and 22.7% for the first and second doses of the vaccine, respectively [82]. The majority of the individuals who were likely at higher risk of MPV infection were not vaccinated, despite the jurisdictional threshold approach. Additionally, racial and ethnic minority groups were less likely to be vaccinated, despite being disproportionately infected by MPV [82,84,85].

Efforts should prioritize increasing immunization rates among populations at higher risk of MPV infection. Continuous and comprehensive surveillance of MPV infection’s geographic distribution and epidemiology is imperative. This surveillance can identify populations with lower immunization rates, even when they are disproportionately affected or considered to be at high risk. It can also reveal regions with low immunization rates among high-risk groups, highlighting the need for intensified efforts in these areas. Data from this surveillance can guide targeted local outreach through health fairs and public service announcements, promoting awareness and health literacy about MPV and the benefits of immunization. Importantly, education should avoid stigmatizing individuals at higher risk, mainly men who have sex with multiple partners [85]. Overcoming misconceptions that MPV vaccination is exclusively for those engaging in promiscuous behavior or based on sexual preference is crucial. Emphasizing that vaccination status remains confidential can address concerns about judgment. Health fairs should offer on-site vaccination, and public service announcements should provide clear information on where individuals can access vaccinations.

LC16m8 is another third-generation smallpox vaccine. It is a live attenuated smallpox vaccine that was developed from Lister, which was a first-generation vaccine composed of live VACV [86]. Attenuation was likely achieved through a single-nucleotide deletion mutation of the *B5R* gene possessed by the Lister strain, leading to a truncated version of a membrane protein [87]. Studies showed that a single vaccination with LC16m8 in cynomolgus monkeys (*Macaca fascicularis*) provided protective immunity against MPV for up to 12 months and led to less severe symptoms of MPV infection when compared to control groups [88,89]. These studies were not able to determine whether immunogenicity from LC16m8 lasted longer than 12 months [88,89]. Clinical trials investigating the immunogenicity and safety of LC16m8 in humans demonstrated sufficient seroconversion 30 days after vaccination, without serious adverse events [90,91]. Future studies should be performed to determine the duration of immunogenicity after LC16m8 vaccination and to further assess its safety profile.

The rapid production and effectiveness of mRNA-based COVID-19 vaccines [91,92,93], as well as the development and Emergency Use Authorization (EUA) of a DNA-based COVID-19 vaccine in India [94], has created inspiration for the development of nucleic-acid-based vaccinations for MPV. mRNA and DNA-based vaccines have been reported to be promising and potentially more advantageous alternatives to traditional virus-based vaccines due to their relatively low cost, rapid manufacturing, and safety, as they are non-infectious and pose no risk of integration into the host genome [95,96]. Additionally, mRNA-based vaccines can be modified in vitro to specifically regulate features such as half-life and immunogenicity [96]. Several mRNA-based MPV vaccines have been developed and employed in animal models. These studies have demonstrated robust immunogenicity in mice that received the vaccines and were exposed to the VACV challenge [97,98,99,100], as well as less severe pathological changes than in control groups [97,98,100]. Interestingly, DNA-based vaccines developed from VACV for protection against MPV had been produced and trialed in animal models years before the COVID-19 pandemic. Non-human primates given these DNA-based vaccines and exposed to MPV challenge developed strong immune responses and were protected from severe disease [101,102]. Nucleic-acid-based vaccines for MPV represent a promising alternative to JYNNEOS that could become especially useful if there is a shortage of JYNNEOS supply. However, nucleic-acid-based vaccines require substantial further research to assess their safety and efficacy in humans.

## 7. Monkeypox Outbreak and Impact on Health Systems

MPV has emerged as a novel threat to the global health systems that are still striving to recover from the devastating impact of the COVID-19 pandemic, especially in low- and middle-income countries (LMICs), due to their lack of financial resources. For many countries, COVID-19 has consumed a major portion of their health budgets, as was evident from how the healthcare systems struggled with inadequate manpower, ventilators, hospital beds, healthcare professionals, and medical equipment [103]. Even though not many monkeypox cases have been reported in LMICs, which might result from a shortage of healthcare professionals, leading to cases being potentially underdiagnosed, the spread of the virus is almost inevitable, and the health systems will be on the edge of collapse once the disease starts to spread. Given how some epidemics and viral diseases, such as polio, dengue, Zika, Crimean–Congo hemorrhagic fever, and others, have resulted in numerous casualties for some nations in the past, it is important to consider a proactive approach and an urgent plan of precautionary measures to control and prevent the continuous decimation by MPV [103,104,105,106].

**Figure 2:**
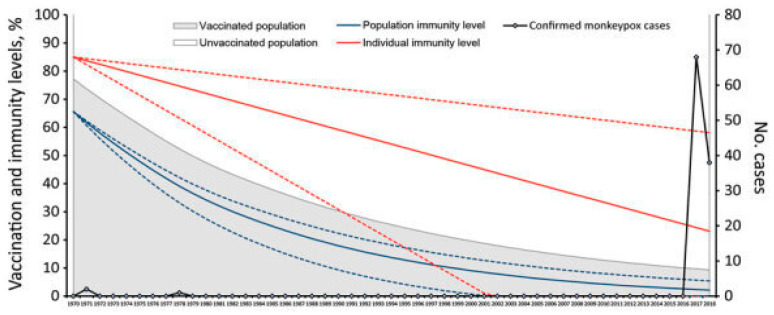
Relationship between population- and individual-level smallpox vaccination and immunity rates and resurgence of MPX cases in Nigeria between 1970 and 2018. Image was retrieved from https://www.ncbi.nlm.nih.gov/pmc/articles/PMC8007331/ [109] (Accessed on 15 October 2023).

Although smallpox vaccines have been shown to be effective against MPV, immunization against smallpox was put on halt after the successful eradication of the disease, leaving a significant proportion of the population in many non-endemic countries susceptible to contracting MPV (Figure 2). Although the exact cause for the re-emergence of MPV has not yet been established, the cessation of smallpox vaccinations after the successful eradication of the disease, leading to waning immunity in the population, might be a possible driver [4,107]. In addition, the emerging cases of MPV in non-endemic regions point towards the negligence of public health emergency alerts and measures to curtail the spread of the disease, despite decades of continuous outbreaks. For example, Nigeria has now reported MPV cases in all regions, while in 2019 the reported cases were confined to the southern parts of the country. The diversion of public health resources and surveillance focus due to the COVID-19 pandemic and Lassa fever outbreaks could be a possible reason for the dispersion of this viral ecological niche [107]. Given the unsuccessful public health measures to confine the disease’s dispersion, the global monkeypox outbreak was declared a public health emergency of international concern by the World Health Organization in July 2022, which is the highest-level alert issued by the UN’s public health body, previously attributed to major global outbreaks of COVID-19, polio, Ebola, and the Zika virus [108].

Strengthening primary healthcare systems is a significant first step to tackling such outbreaks. Countries need to be equipped with operational disease surveillance systems, trained public health workforces, and a prime emphasis on a “One-health” approach to deal with viral outbreaks [110]. Many countries are not yet equipped with appropriate diagnostic facilities, and samples from infected patients often have to be sent abroad for testing, which further increases the possibility of the spread of disease due to possible exposure and contamination [103]. Establishing in-house diagnostic facilities and rapid testing capabilities at the local level is imperative. This approach not only minimizes the risk of disease spread due to sample transportation but also ensures a swift response to suspected cases. The COVID-19 pandemic has enhanced the genomic sequence testing capacities in many countries, and genomic sequencing would be beneficial to identify the chain of infection of MPV cases during an outbreak. However, genomic sequencing is expensive, time-consuming, and does not add value to the identification of emerging cases, given that MPV is a DNA virus with around 200,000 nucleotide bases, which is six times more than what we saw in SARS-CoV-2 [110].

International collaboration is a linchpin of success in this endeavor, allowing for the sharing of information, resources, and best practices. This cooperation should extend to supporting low- and middle-income countries in building their capacity for effective surveillance and testing. Adequate funding and resource allocation from governments, international organizations, and donors are essential to sustain these vital public health initiatives. By embracing these strategies, countries can bolster their readiness to detect, control, and ultimately mitigate the impact of MPV outbreaks, ensuring the health and wellbeing of their populations on a global scale.

## 8. Lessons Learned from the COVID-19 Pandemic

The COVID-19 pandemic has bestowed us with several important lessons pertaining to the management of emergent viral outbreaks, such as the recent MPV emergency, that cannot be ignored. The most important lesson revolves around preparedness for such emergencies. The COVID-19 pandemic began in December 2019 and has so far been associated with nearly 7 million deaths worldwide [111,112]. The first vaccine for COVID-19, the Pfizer-BioNTech COVID-19 Vaccine, was made available for widespread use after FDA approval via EUA in December 2020, one year after the start of the pandemic [113]. Although tireless work went into the production of this vaccine and it was produced relatively quickly, faster production and utilization of vaccines may prevent many deaths and reduce morbidity in such viral outbreaks [111]. Additionally, the surfacing of several variants of COVID-19, namely, Omicron and Delta, provides a rationale for closely monitoring and preparing for the evolution of MPV and other viruses that threaten public health [114,115,116,117]. Viral evolution may result in more virulent strains, as well as strains that are not effectively identified by current diagnostic modalities or neutralized by existing vaccines. Monitoring for MPV’s evolution can be performed following a protocol similar to one used in a study to assess the genomic variability of MPV in the Democratic Republic of Congo [118]. Surveillance for active monkeypox disease should be carried out by local clinicians and public health agencies. These entities should announce that individuals should seek medical attention if they are experiencing symptoms that are consistent with MPV infection and then report cases that they encounter. MPV infection should be confirmed in these cases by quantitative polymerase chain reaction (QPCR). With the consent of each patient, a scab or vesicular fluid should be directly sampled and undergo genomic sequencing. Comparing and analyzing the genomic sequencing of each sample and correlating the results with disease severity, degree of transmissibility, and geographical pattern can provide information regarding potential MPV mutations and associated virulence [118]. Preparedness for future viral outbreaks should be bolstered by allocating resources to implement this monitoring method by improving and expanding population surveillance, diagnostic testing, and laboratory viral genomic sequencing, as well as developing a standardized protocol and system for immediate laboratory testing and vaccine production when encountering a new MPV strain. This system can result in the rapid production of updated vaccines that are more effective at protecting against new MPV strains than pre-existing vaccines, which can contribute to reducing the spread and further evolution of these strains. Additionally, stockpiles of vaccines that are currently available should still be created to serve in the event that an outbreak occurs and updated vaccines cannot be produced fast enough, so that there is still some level of protection against MPV.

It has been suggested that the COVID-19 pandemic began because of transmission of the SARS-CoV-2 virus from animals to humans at several markets in Wuhan, China that sold wild animals [119,120]. Monkeypox is also a zoonotic disease, and past MPV cases and outbreaks have been determined to be caused by transmission from wild animals, stemming from activities such as hunting, skinning, cooking, processing, eating, or transporting these animals [121]. The world should learn from these incidents in an effort to reduce and prevent future MPV outbreaks originating from animals. Measures should be taken to reduce the frequency of activities that expose individuals to transmission of MPV from wild animals, including passing and enforcing laws that prevent the handling, hunting, trading, and cooking of wild animals that are suspected to be possible MPV reservoirs in areas where MPV is spreading. If individuals still insist on handling these animals, they should be encouraged to wear sufficient personal protective equipment when handling them, sanitize all involved surfaces and equipment, and thoroughly cook the animals before eating [121].

Another crucial lesson learned from the COVID-19 pandemic that will prove useful in dealing with global emergencies is the importance of early, continuous, and clear communication and guidance from public health agencies during public health emergencies. The politicization of public health agencies’ conduct during the early stages of the pandemic resulted in the widespread circulation of medical misinformation and unclear and contradictory messages regarding the course of the pandemic and recommendations for the public. As a result, there was diffuse confusion and distrust of public health agencies, which likely contributed to the increased spread of COVID-19, as many individuals were not aware of or compliant with measures that could have been taken to reduce the viral spread [122]. Public health agencies should be free from politicization in the future to provide people quickly and accurately with clear, evidence-based recommendations and education regarding best practices during a public health emergency. It is also important to obtain information from local public health officials, as they will know their community the best and they will know whether or not the national guidance applies to them. Additionally, regional responses can be quite helpful to have the hospitals in a particular community communicating and collaborating with one another. During the COVID-19 pandemic, each state had its own response, and there were varying levels of success. It may also be helpful for national and local organizations to make use of social media, since this is a great way to quickly spread information and dispel misinformation. Misinformation is less likely to spread if there is good information being disseminated to the community by reliable resources. As part of the preparation for the next crisis, it is important for the community, regional, and national leadership to take steps to gain the trust of the population now. If there is already trust in the leadership before the crisis occurs, it is more likely that the people will follow them during the crisis. Hospitals should also be aware of their surge capacity. If this virus should start spreading rapidly through a community, the local hospitals will have to address the rise in cases. The lack of preparedness and communication during the COVID-19 pandemic should also be used as an opportunity to continuously educate the public about methods to not only manage future viral outbreaks but also prevent them from happening in the first place [123]. Public health agencies should disseminate clear, evidence-based information to enhance public health literacy on MPV infection. This should include educating the public on infection signs, transmission methods, risk factors, vaccination, and hygiene practices, such as handwashing, mask-wearing, sanitation, and avoiding direct contact or item-sharing. Guidance should also address the necessary steps when infection is suspected: seeking healthcare, isolating, and notifying close contacts for timely vaccination within 14 days of exposure to minimize the disease’s impact. Elevating public awareness of MPV and preventive measures will significantly curtail its transmission and future outbreaks [124].

## 9. Conclusions

The emergence and rapid spread of monkeypox virus (MPV) across the globe presents a complex and evolving public health challenge. MPV, despite its lower fatality compared to smallpox, poses a substantial threat, particularly in regions where cross-protective immunity from smallpox vaccination has waned. The current global outbreak, with its atypical presentation, epidemiological variations, and the emergence of new cases in non-endemic regions, underscores the need for immediate and concerted action.

The development and distribution of effective vaccines, such as JYNNEOS, have shown promise in preventing and mitigating MPV infections. However, there remain critical uncertainties regarding dosing, safety, indications, and contraindications that require further research. Nucleic-acid-based vaccines offer a potential alternative, especially in situations of vaccine supply shortages. These vaccines, inspired by the success of COVID-19 mRNA vaccines, require rigorous evaluation for safety and efficacy.

Lessons from the COVID-19 pandemic emphasize the importance of preparedness, clear and transparent communication, and a coordinated global response. Public health systems must strengthen their capacity to detect, respond to, and control emerging viral threats like MPV. This should include bolstering disease surveillance, training healthcare professionals, and adopting a holistic “One Health” approach. Genomic sequencing can aid in understanding the virus’s evolution and spread.

Furthermore, the impact of public health emergencies on healthcare systems, especially in low- and middle-income countries, cannot be underestimated. These systems already have limited resources and are likely strained by the COVID-19 pandemic, so they need proactive measures to prevent further disruption. Lastly, addressing the monkeypox virus outbreak and future viral threats demands a multifaceted approach encompassing research, vaccine development, strengthening healthcare systems, and global cooperation. The current crisis serves as a stark reminder of the need for sustained vigilance, investment in public health infrastructure, and a commitment to science-based solutions for safeguarding global health.

## Figures and Tables

**Table 1:** Top 15 countries by total confirmed cases (1 January 2022–30 September 2023).

Country	Sum of Total Confirmed Cases
United States of America	30,636
Brazil	10,967
Spain	7580
France	4154
Colombia	4090
Mexico	4062
Peru	3812
United Kingdom	3782
Germany	3703
Canada	1496
China	1484
Chile	1442
Netherlands	1274
Argentina	1130
Portugal	1050
**Grand Total**	**80,662**

Data were retrieved from the World Health Organization (https://worldhealthorg.shinyapps.io/mpx_global/_w_ab623397/#section-global-deaths-1, accessed on 1 October 2023).
